# Acceptability of dietary and physical activity lifestyle modification for men following radiotherapy or radical prostatectomy for localised prostate cancer: a qualitative investigation

**DOI:** 10.1186/s12894-017-0284-5

**Published:** 2017-10-10

**Authors:** Lucy E. Hackshaw-McGeagh, Eileen Sutton, Raj Persad, Jonathan Aning, Amit Bahl, Anthony Koupparis, Chris Millett, Richard M. Martin, J. Athene Lane

**Affiliations:** 1 University of Bristol, NIHR Biomedical Research Centre - Nutrition, Diet and Lifestyle Theme, Level 3, University Hospitals Bristol Education Centre, Upper Maudlin Street, Bristol, BS2 8AE England, UK; 2 University of Bristol, Bristol Medical School: Population Health Sciences, Canynge Hall, 39 Whatley Road, Bristol, BS8 2PS England, UK; 3 Bristol Urological Institute, Southmead Hospital Bristol, Southmead Road, Westbury-on-Trym, Bristol, BS10 5NB England, UK; 4 Freeman Hospital, Freeman Road, Newcastle upon Tyne, Tyne and Wear, NE7 7DN England, UK; 5 Bristol Haematology & Oncology Centre, Horfield Road, Bristol, BS2 8ED England, UK; 6 Member of the NIHR Biomedical Research Centre - Nutrition, Diet and Lifestyle Theme Prostate Cancer Patient and Public Involvement Group, Bristol, England, UK

**Keywords:** Clinical implications, Intervention, Interview, Lifestyle, Nutrition, Physical activity, Prostate cancer, Qualitative, Radical prostatectomy, Radiotherapy

## Abstract

**Background:**

The experience and acceptability of lifestyle interventions for men with localised prostate cancer are not well understood, yet lifestyle interventions are increasingly promoted for cancer survivors. We explored the opinions, experiences and perceived acceptability of taking part in nutritional and physical activity interventions amongst men with prostate cancer and their partners; with the ultimate plan to use such information to inform the development of nutritional and physical activity interventions for men with prostate cancer.

**Methods:**

Semi-structured interviews with 16 men, and seven partners, undergoing curative surgery or radiotherapy for prostate cancer. Interviews explored experiences of lifestyle interventions, acceptable changes participants would make and perceived barriers and facilitators to change. Interviews were thematically analysed using the framework approach.

**Results:**

Men were frequently open to lifestyle modification and family support was considered vital to facilitate change. Health beneficial, clinician endorsed, understandable, enjoyable interventions were perceived as attractive. Barriers included ‘modern’ digital technology, poor weather, competing commitments or physical limitations, most notably incontinence following radical prostatectomy. Men were keen to participate in research, with few negative aspects identified.

**Conclusions:**

Men are willing to change behaviour but this needs to be supported by clinicians and health professionals facilitating lifestyle change. An ‘intention-behaviour gap’, when an intended behaviour does not materialise, may exist. Digital technology for data collection and lifestyle measurement may not be suitable for all, and post-surgery urinary incontinence is a barrier to physical activity. These novel findings should be incorporated into lifestyle intervention development, and implemented clinically.

## Background

Approximately one in eight men will be diagnosed with prostate cancer in the UK, and it is the most common male cancer in the Western world [1]. Cancer risk and disease progression have been linked to a variety of lifestyle factors and it has been estimated that up to one third of all cancers may be attributed to poor diet, lack of physical activity and obesity [2].

Physical activity and nutrition are thought to benefit physical and psychological health in cancer survivors [3]. Accumulating observational evidence suggests that physical activity and a healthy diet improve overall survival in men with prostate cancer [4, 5], yet most men with prostate cancer do not spontaneously alter their diet or physical activity following a diagnosis [6, 7].

Due to this lack of spontaneous change, much research, including randomised controlled trials, have attempted to influence lifestyle changes in men with prostate cancer; however, these are often underpowered, at high risk of bias, inadequately reported or of short duration, prohibiting reliable conclusions from being drawn [8]. For example, in a systematic review of dietary, nutritional and physical activity interventions in men with prostate cancer half of the 44 trials identified had high risk of bias for sequence generation and blinding of participants, and 21 of the 44 failed to report whether the trial was sufficiently powered [8].

Previous qualitative research to evaluate the experiences of men with prostate cancer, and their attitudes towards participating in lifestyle behaviour change interventions, concluded that men were often positive about making such changes, were optimistic about participation in an intervention and expressed a desire to ‘give something back’ by participating in research [9]. Group interventions were favoured over individual activities [10], but the shock, distress and anxiety of a diagnosis led to sometimes strained relationships with partners [9] and it was (perhaps surprisingly) often not desired that partners took part in an intervention alongside the men [10]. This is in contrast to studies supporting the efficacy of couple based interventions [11]. Previous research exploring lifestyle interventions have often included men undergoing watchful waiting [9] and androgen deprivation therapy [10]. Our focus was on men who had previously undergone, or were currently undergoing, radical prostatectomy or radiotherapy for localised prostate cancer. This is a population of men who are expected to survive after their prostate cancer treatment for a long period of time, but who nevertheless have a risk of either prostate cancer recurrence or a second primary cancer [12]. These survivors will be at risk of competing medical comorbidities such as cardio-respiratory mortality [13] and in addition to the cardio-metabolic effects of androgen deprivation therapy or later salvage treatments and so might benefit from making long-term dietary and physical activity changes [3].

We explored the opinions, experiences and perceived acceptability of taking part in nutritional and physical activity interventions amongst men with localised prostate cancer, who had previously undergone, or were currently undergoing, radical prostatectomy or radiotherapy, and their partners; with the ultimate plan to use such information to inform the development of nutritional and physical activity interventions for men with prostate cancer [14].

## Methods

### Participants and recruitment

Men were recruited from one hospital urology outpatient clinic in England between July and December 2013. Using a convenience sampling approach [15], consecutively eligible men with clinically localised prostate cancer, who had recently undergone robotic radical prostatectomy or were undergoing radiotherapy, and with sufficient understanding of the English language, were introduced to the researcher by the clinicians. Men thought not to be suitable for interview by their Urology health care team were excluded. Those who agreed to participate were invited to invite their partner, where applicable, to take part in the interview process. This was optional and reasons for partners being included, or not, were not explored further.

### Procedures and data collection

Interviews were conducted by either LHM (*n* = 13) or ES (*n* = 10). Participants were interviewed individually, except where men and their partners chose to be interviewed together (*n* = 5). The majority (*n* = 16) were conducted at the participant’s home, or within a private location at the urology clinic. However, some (*n* = 7) took place via telephone at the participant’s request. Interviews lasted from between 26 and 95 min (mean 49 min).

Following a general introduction, an example 6 month lifestyle intervention was described by the researcher, where men would be asked to walk briskly for an additional 30 min, 5 days a week, alongside either taking a daily lycopene supplement or increasing fruit and vegetable intake and reducing dairy milk intake. The semi-structured interviews included the following topics: previous experience of nutritional and physical activity interventions, lifestyle behaviours that the men would and would not be happy to change, expected gains and costs of participating in an intervention, and perceived barriers and facilitators to change. An interview schedule, developed through reviewing previously discussed existing literature, acted as a guide, but allowed the researcher flexibility to examine the participants’ perspectives and gain an insight in to their thoughts and feelings. Additionally, all participants provided a range of demographic data.

### Analysis

Interviews were digitally audio-recorded and transcribed verbatim. Thematic analysis was conducted using the framework approach by LHM [16]. Transcripts of five participants (22%) were analysed by both LHM and ES to ensure validation triangulation of themes. NVivo (NVivo10, QSR International, 2012) was used to assist with the analysis. Here we present only the analysis relating to behaviour change and intervention development; other topics will be reported elsewhere, for example patient’s views and experiences of advice provision by health care professionals [17]. The COREQ (COnsolidated criteria for REporting Qualitative research) Checklist was used to guide the design, implementation and analysis of this research.

The NHS North West - Lancaster NRES Committee approved the study (13/NW/0228). All participants provided fully informed consent.

## Results

In total 16 men with prostate cancer (mean age 67 years; range 53 – 79) and seven partners (mean age 65 years; range 47 – 77), were interviewed. Aggregated demographic information is shown in Table 1 and individual level characteristics are illustrated in Table 2. Twelve men had surgery (on average 6 months prior to the interview, range 2 – 15 months) and 4 were undergoing radiotherapy as treatment for their prostate cancer. Seven men declined the invitation to participate, equating to a recruitment rate of 70% (16/23). The following reasons were given for not wanting to participate; unable to be contacted (*n* = 2), too overwhelmed by cancer experience or incontinence (*n* = 2), felt too unwell (*n* = 1), simply did not want to (*n* = 1) and finally ‘concerned about computer hacking’ (*n* = 1).

**Table 1:** Demographic characteristics of the men with prostate cancer and their partners

	Men with prostate cancer: 12 surgery; 4 radiotherapy	Partners: 3 surgery; 4 radiotherapy
Mean age in years (range)	67 (53–79)	66 (47–77)
Ethnicity	White British (100%)	White British (100%)
Gender	Male (100%)	Female (86%)
Highest level of education	Attended school to 16 years or less (60%)	Attended school to 16 years or less (57%)
Completed university	2 (13%)	1 (14%)
Marital status	Married (81%)	Married (86%)
Occupation	Retired (50%)	In work (75%)
Drinking alcohol	Drink on 1 or 2 days per week (31%)	Drink almost every day (33%)
Smoking	Non-smoker (88%)	Non-smoker (86%)

**Table 2:** Participant ID and key individual characteristics of the men with prostate cancer and their partners

Participant ID	Age group	Relationship	Treatment	Duration since end of treatment at time of interview
Patient1	71–76	Married	Surgery	4–6 months
Patient2 & Partner1	65–70 & 59–64	Married	Surgery	1–3 months
Patient3	65–70	Not married	Surgery	10–12 months
Patient4 & Partner2	NK & 71–76	Married	Surgery	> 12 months
Patient5	71–76	NK	Surgery	> 12 months
Patient6 & Partner3	65–70 & 65–70	Married	Radiotherapy	< 1 month
Patient7	53–58	Not married	Surgery	1–3 months
Patient8	71–76	Married	Surgery	1–3 months
Patient9	59–64	Married	Surgery	1–3 months
Patient10 & Partner4	59–64 & 65–70	Married	Surgery	1–3 months
Patient11	65–70	Married	Surgery	7–9 months
Patient12	65–70	Married	Surgery	4–6 months
Patient13 & Partner5	77–82 & 77–82	Married	Radiotherapy	< 1 month
Patient14 & Partner6	65–70 & NK	Married	Radiotherapy	< 1 month
Patient15 & Partner7	59–64 & 47–52	Not married	Radiotherapy	< 1 month
Patient16	65–70	Married	Surgery	1–3 months

All participants were generally very positive about the proposed 6 month lifestyle intervention. Five main themes emerged: three related to motivations, facilitators and barriers to change; one related to research participation; and one related to lifestyle intervention characteristics.

### Motivations for change

Motivations for change were discussed and were often supported by a partner. Shock at the time of diagnosis were frequently discussed, and on many occasions, this had resulted in participants taking stock of their current lifestyle behaviour, identifying that change may be necessary.

*If it meant that I could prolong my life to a certain extent, without having to suffer, I would most probably consider it….If it meant I had to cut out certain food, because they’re the ones that do the problem to my body, then I would. I wouldn’t hesitate. Because if it’s doing damage to me, that’s it (Patient14-RAD)*

*If somebody said to me, “You’ve got to walk from here to the ring road” which is probably five minutes, ten minutes’ walk, a fast walk, “And back every day, otherwise you’ll be dead in six months” I would do it without hesitation (Patient6-RAD)*

It should be noted that both men quoted here suggested that they were motivated by reducing mortality and suffering, not specifically by improving health and wellbeing.

### Facilitators of change

#### Family support

Almost all men discussed the benefits of family or social support and as the majority (81%) of the men were married; subsequently, many suggested that their wife would either join in with an activity or assist with preparation of food.

*I think [wife’s name] would be very encouraging because you know, she thinks I don’t eat healthily enough, so I think she would, there wouldn’t be a problem in that sense, if I suddenly went vegetarian or I had to [eat] fish….if I was doing this I would do the same and eat the same as [wife’s name], because I think that would probably help if we were both eating the same….she’d love it, if you know what I mean. She’d have a right grin on her face I think as she’s cooking it for me (Patient6-RAD)*

The importance of involving a partner in an intervention to change behaviour was discussed by many, independent of whether a partner was present in the interview. It was generally felt that the support of a partner would be a facilitator to successful implementation.

*Interviewer: So what about your typical diet, what would you say you eat ....?*

*Patient: Well [Laughter]*

*Partner: Do you want to answer that?*

*Patient: I eat whatever I’m given (Patient4 & Partner2-SUR)*

This highlights the need to ensure that partners, where applicable, are involved in all elements of an intervention. For example, by inviting the partner along to a recruitment appointment, and ensuring that they are aware of the specific nature of the intervention, particularly for dietary elements. This may not be the case for all; however, in this specific population, the partners, often a wife, were key to the purchase and preparation of food.

Others discussed their partner, children, or friends providing emotional, informational, tangible support (eg. providing financial assistance or material goods) [18]. Such support may involve making healthy snacks for work or no longer buying unhealthy food for the home and it was believed that this social support would help promote success. One participant discussed the impact of his recent divorce, and subsequently being less physically active, as this was something that he and his former partner had previously done together.

*It’s extremely difficult for an isolated individual to make that [behaviour] change (Partner7-RAD)*

#### Health gains and clinical advice

Personal motivators to change varied and included weight loss or expectancy to improve general health. Weight gain was often discussed as a stimulus for carrying out physical activity or dietary interventions, especially if a health care professional had raised the issue.

*A couple of years ago, I don’t know how long it is, two, maybe three years, he went to have a health check at the surgery. He came back horrified because he said, “The nurse told me I’m obese.” And that really upset him….that affected him. He then watched what he ate….he stopped having breakfasts and he wasn’t eating anything….I think he was frightened of being fat (Partner3-RAD)*

If a current behaviour was seen as potentially life threatening, this was also reported to be a personal motivator for change, with enhanced impact if recommendations for behaviour change were delivered by a health care professional.

*“Well, I mean, you’re… You tend to be in the hands of, of your consultants and, and the team, so if they, if they tell you something should be done for your benefit, then you probably do it” (Patient3-SUR)*

*If you take alcohol, if [health care professional] said …“You will not survive another five years if you continuing drinking one unit a day” or something. I’d have to make a change (Patient4-SUR)*

#### Rationale for change

Many men explained that knowing the reasons for lifestyle changes or understanding the underlying science, or how the specific change would benefit them, would help facilitate change.

*I suppose if you’ve got a, a definite link between; “If you eat that, then…” You know, in 10 years’ time, such and such might happen, then obviously it makes you think (Patient3-SUR)*

*If someone had turned round and said, “Look, these are the benefits of changing your diet,” I would have done. But you know … I’d have to be shown and say, “Right, this is the situation, this is - if you change and have these, this would be an improvement,” that’s fine I would do that (Patient12-SUR)*

#### Anticipated enjoyment of lifestyle changes

Increased enjoyment of a behaviour was thought to encourage perseverance; likewise, it may prevent uptake if the activity is perceived to be boring or unenjoyable. For example, some suggested that they would be more likely to participate if they were walking for a purpose, such as to buy a newspaper.

*My physical activity has always been doing things, going with dogs walking, diving, doing things in that way, rather than just going and work on a treadmill, that would bore… I wouldn’t… If you asked me to do that I’d say, “No.” (Patient12-SUR)*

Others discussed that liking the taste of what they had been asked to consume would make them more likely to continue with it in the longer term.

### Barriers to lifestyle change

Although men were generally positive about changing their behaviour and specifically in relation to the proposed intervention, there were several potential barriers to change.

#### Poor weather

Weather was discussed by many of the men and their partners. It was often implied that despite enjoying an activity, poor weather conditions could prevent the activity taking place, for example:

*If it was raining, I’d sit on my backside [laughter] (Patient2-SUR)*

*I cycle when it’s dry (Patient8-SUR)*

*It’s a cold time of year, obviously, which limits you a little bit (Patient16-SUR)*

During shorter winter days, some of the men suggested that they may be less inclined to go out walking; however, others talked about working outside, and this not being a problem for them; it was explained that in poor conditions they would carry out household chores or another task, and then do additional walking when the weather improved.

*If it’s pouring with rain, I would say no, but the thing is….you do other things….other weeks may compensate for your bad weeks….if I went out Monday, and the weather’s bad on Tuesday, we may go somewhere Thursday, or Wednesday (Patent14-RAD)*

This barrier could be seasonal; however, in countries where weather is often unpredictable it should be considered.

#### Urinary incontinence

Incontinence is a well-established potential side effect of radical prostatectomy and almost all men who had undergone surgery identified incontinence as an important barrier to physical activity. This was not discussed by those who had undergone radiotherapy.

*The thing is, when you’ve had the operation walking is a problem, initially. Until….you can control yourself a bit better. Then walking is a, it acts like a pump, you know what I mean? So you’d have to wait….before you were quite confident walking a long way anyway (Patient5-SUR)*

*I think perhaps I could do a bit more, bit more walking, but at the moment I’m still got problems with this, the incontinence so I don’t like to go too far (Patient9-SUR)*

Men described fear of leakage whilst participating in physical activity as often debilitating and causing embarrassment.

*I’m still incontinent … I wear pads and I do find that if I’m walking around I obviously leak a bit and I can feel myself getting, you know, the pads getting full, if you like. And I have to then go and then sort it out (Patient10-SUR)*

Ways to overcome issues surrounding incontinence were discussed with the participants, for example finding well-fitting and comfortable pads, exercising when it was darker, so leaks were less noticeable, or mastering pelvic floor exercises, which can help to control urinary incontinence.

#### Time pressures and overall health

A further barrier to making changes to behaviour could be competing time interests, as illustrated below.

*That’s a problem, you know with all the activity wot I do now….some days, some days I probably would [be able to do additional walking], but won’t be every day I shouldn’t think, because it’s a time factor, it’s a time factor, to put in with my schedule wot I do on a week (Patient1-SUR)*

Although others suggested that they would be prepared to incorporate activities like walking into their routine.

*The only cost would be would you’d have to organise your day, your time to fit it in. You could either do it first thing in the morning or when you get home from work. Go for a walk, you know, before lunch- before dinner or probably try and fit in a bit more exercise at work, a bit more walking in at work. I could probably rearrange a few things so that I could do a bit more, bit more walking at work (Patient9-SUR)*

Other unrelated health conditions such as knee replacements, arthritis, bowel problems, stroke and heart attacks were discussed as individual physical barriers to making changes.

*It’s alright if you’re in, in reasonable physic… Good physical health, but if you’re not then these things become more difficult (Patient3-SUR)*

*Partner: The only thing I’m thinking is, is your leg going to let you do that? Not because he wouldn’t want to do it, but I’m not sure if your leg would let you do that.*

*Patient: No, not going too fast, that’s the problem. I’ve had a new knee in there (Patient13 & Partner5-RAD)*

### Research participation

The overall opinion portrayed by the men was that once they had committed to participating in the research, then they would do whatever was asked of them. Some referred to this as being stubborn, others as being determined to support research, but it was implied that once they agreed to be involved, they would not change their mind.

*Interviewer: So if you were in our trial and we said to you, “Please drink three cups of green tea a day”?*

*Patient: I’d do it. ‘cause you’ve asked. I always do what I’m told, almost….Well just to say that I’m doing it for this particular purpose. It’s a good enough reason for me to do it (Patient10-SUR)*

This may be due to an altruistic wish to help provide research data, an intention to improve one’s own or other people’s health, having a specific interest in the study or simply a desire to see a ‘project’ through once committed to it.

*Interviewer: If we had asked you to walk an additional 30 minutes at a brisk pace every day, how would you feel about that?*

*Patient: Yes… Well I suppose if it was part of a trial, I’d, I’d do it for the, sort of, esoteric act of being part of a trial (Patient3-SUR)*

*I’m quite open to try anything, because….this is really an experiment that may benefit people in the future….to really benefit the people for the future as a younger generation….I thought if it could help someone in future years, it’s well worth it (Patient14-RAD)*

We also explored any anticipated negative aspects of participation in an intervention trial. Some men mentioned specific foods that they would find challenging to eat (one example given was curry) or would find hard to give up (such as cheese).

*If I tried it and I liked it, I’d eat it, if I didn’t like it, I wouldn’t eat it (Patient1-SUR)*

One participant explained that despite being committed, if asked to do something he did not wish to, it would be unlikely to happen.

*Interviewer: If it's something you didn’t like, is there any way to get you to do it?*

*Patient: You’d have to work hard [laughter]….*

*Interviewer: Are there any techniques we could use to get you to do something that you weren’t keen on?*

*Partner: No, because I think, he would sort of, “Do you want to..?” “No”. “Right, okay, I’m off, then.”*

*you’ve always been like that.*

*Patient: Always been like it, I think (Patient2 & Partner1-SUR)*

The majority of men, and their partners, described busy lifestyles, including carrying out odd jobs, sitting and reading or ‘pottering’, and some were still in full time work. Generally, however, participants illustrated that it would not be a sacrifice, implied that they would be happy to make changes, and would try to find time to do so.

*If you want to do something you always fit it in somewhere….you should hopefully lose a bit of weight, feel better, you know, so there’s no real losses except a bit of time (Patient12-SUR)*

### Lifestyle intervention characteristics

#### Research data collection methods: Internet, phone and paper

There was a general mistrust regarding e-technology and online data collection.

*Interviewer: So what about having records like that, on that kind of technology? Would that be okay?*

*Patient: I don’t really know how these things work, but I am worried. I had a [company] phone and [the company] are beginning to worry me a bit ‘cause they, they might well intercept and take all that information as well. [They] do take a lot of information in your transmission of data….*

*Partner: I don’t use the computer at all. I think it’s intrusive and dangerous (Patient4 & Partner2-SUR)*

This sentiment was further illustrated by lack of use of social media, which was recommended for younger people, and was seen to be too intrusive.

*I think it would appeal to … the younger people. I mean, and I’m personally not on Twitter or Facebook (Patient3-SUR)*

*I don’t take part in Twitter or, or any of those. They’re for children aren’t they, really? (Patient4-SUR)*

*That’s a bit personal if you ask me. I'm not a Facebook person; I don’t understand it, I don’t understand it at all. I know how it works, but for me it just baffles me. I can’t understand why somebody would write on Facebook, “Had a hard day at work, now I'm sat down drinking a cup of tea.” I'm thinking, “Okay, fair enough. Why do you want to say that?” But people do, so it’s just me being old fashioned I suppose (Patient7-SUR)*

Some participants regularly used their mobile phone for sending text messages, kept it turned on and would be comfortable to use it for research purposes. However the majority of men said that they either did not own a mobile phone, they rarely turned it on, or they would not know how to respond to a text message.

*Interviewer: Do you use electronic devices, at all? Do you have a mobile phone?*

*Patient: Yes, with difficulty. I’ve got a mobile phone….For emergencies only*

*Partner: He never uses it….*

*Patient: My phone’s from the Ark. I dial, and I can text*

*Partner: It might take him several days to text [laughter]*

*Patient: It’s not something that I am partial to doing. It’s easier to go, “Hello?”….I’ve never got the phone on….I don’t very often pick my phone up, you wouldn’t get an answer. That’s basically the bottom line (Patient2 & Partner1-SUR)*

Participants were more positive about computers and email, appearing comfortable to open and reply to an email, download attachments and use a printer. Further to this, many mentioned that neighbours, or children, would be on hand to help out if necessary.

*Oh, I use a computer, no problems at all with a computer, and you know, save paper, do it on the computer. I think that would be a good idea (Patient6-RAD)*

Some participants did not have, or express a desire for, access to a computer or the internet. It was noted, that for the majority of the men, their preferred method of contact was via the post.

*I think I’d rather have a booklet that you fill in. Like, you know, weekly, daily or... something you can actually write in (Patient5-SUR)*

It should be noted that there did not appear to be any demographic or other differences between those who responded positively or negatively to e-technology.

#### Group versus individual interventions

Those men in favour of individual physical activity interventions enjoyed the peace and quiet of being alone, or believed that others could slow them down.

*I think I probably would be able to motivate myself. But probably my wife would probably join in with me. She used to do a little bit of running, a little bit of running before with me, not as much, but she would probably be receptive to the idea. She’d probably say, “Yeah, I’ll come along with you” (Patient9-SUR)*

Alternatively, those who preferred a group intervention felt that other members could motivate them, as well as providing social support.

*If you did it as a, as a group activity. You could join a walking club or… Or, I think the medical practice here runs a sort of, a weekly, healthy walking morning or something….you get the, sort of, support of others. It’s a, it’s a mutual support organisation, isn’t it, when you join something like that? (Patient3-SUR)*

In this particular population, participants tended to favour individual interventions, yet with added spousal or other social support.

#### Dietary modification through supplements

Some men were positive about a daily supplement; feeling that it was an easy, efficient way to provide additional nutritional content to their daily diet.

*Interviewer: Do you take any supplements currently, either of you?*

*Partner: You take cod liver oil...*

*Interviewer: So you’re quite comfortable taking supplements?*

*Patient: Yeah.*

*Partner: Oh yeah (Patient13 & Partner5-RAD)*

In contrast, others disagreed with taking supplements, and would not choose to do so in their general diet. There often appeared to be a sense of disbelief at their efficacy.

*Eating a more of a natural food like tomatoes, I’d be more inclined to do than taking supplements. I try and avoid pills when I can (Patient3-SUR)*

*As for supplements, I think they’re a total waste of time personally….it’s all about intake again. You know, like a lot of people I know take garlic, but if you eat three or four garlic cloves a week, and that’s a lot more than what a tablet a day would do or a capsule a day would do, you know? It’s all about intake and volume. And I’d rather eat the food what gives you those vitamins rather than take supplements (Patient11-SUR)*

However, all men reported that if they were randomised to a supplement in a research study, they would try to incorporate it into their daily routine.

## Discussion

Men with localised prostate cancer, who were undergoing or had completed radical treatment, were generally open to lifestyle modification and were motivated to engage in positive health behaviours. This willingness may result from disbelief, shock or fear at diagnosis and the findings support previous literature, whereby a cancer diagnosis can result in positive behaviour change [3, 9, 10]. Receiving a diagnosis of prostate cancer is considered to be a potential ‘teachable moment’: a naturally occurring health event thought to motivate individuals to spontaneously adopt risk-reducing behaviours [19]. Our findings further substantiate the concept that the period following a cancer diagnosis may be an ideal opportunity to engage with patients to implement diet and physical activity interventions [20].

The men in this study identified the importance of family and friends in motivating them to embrace change, in line with previous findings [21]. As such, when planning behaviour change interventions it is imperative that men taking part are made aware of what additional support is available and where to seek it. Additionally, behaviour change interventions should be tailored, and enjoyable, so that an individual has personal motivation to change; although this may not always be possible for all individuals. Supplements can be an efficient way to enhance the positive nutritional elements that are consumed, where an individual may not like or be able to access a food type [22]. Our findings suggested that knowing that there is an evidence based reason to change, may help assist behaviour change maintenance, particularly if the proposed change is already acceptable to the individual.

Increasingly, research data are being collected using electronic methods, such as web based surveys, text message or social media [23–25]. This is especially true with nutritional data [26]. Engaging the target population with suitable data collection methods is essential, as is avoiding social media if it does not appear to be acceptable to the target population. This is of importance, as many new intervention programmes have a strong technological slant and not considering the accessibility or acceptability to the particular target population may compromise their effectiveness. As technology develops, it could be easy to assume that all research should be collected in such a manner and that research populations would prefer the most high-tech methodologies. However, this may not always be true, as illustrated by many of the current sample preferring to respond to paper based questionnaires. One must always be mindful of an individual’s level of understanding, acceptance and ability to use the technology in question.

Identifying and finding solutions to potential barriers, such as poor weather, competing time commitments or physical limitations such as incontinence, prior to them arising, will make these barriers easier to overcome and less likely to reduce compliance. For example, poor weather could lead to reduced physical activity; however, suggesting participation in indoor physical activities, or swapping the day that the activity is completed on, to avoid poor weather, yet still reaching the week’s intended activity levels, could prove successful. To alleviate incontinence related barriers, well-fitting and comfortable pads could be sourced or men could be supported in the practice of pelvic floor exercises; this could reduce the likelihood of incontinence being a barrier to change.

Elements of the current findings are in line with previous literature derived from men undergoing androgen deprivation therapy [10] and watchful waiting [9], such as the level of shock or disbelief experienced at diagnosis, being motivated to make changes to lifestyle behaviours and having a desire to engage in research. However differing and novel findings emerged from our target group of men undergoing radical prostatectomy or radiotherapy. Men were encouraging of partner support and welcomed input from friends and family, with a preferred emphasis for tailored individual interventions. The use of electronic methods to collect data was suggested to be a potential barrier to participation or change in some men, but not all, as were physical limitations, such as incontinence or underlying unrelated health complaints. Endorsement for change by clinicians was seen to be a motivator for change, along with understanding the rationale behind the suggested change.

Men with prostate cancer demonstrated great intention to commit to research, with few sacrifices identified. It should be considered that social desirability response bias may have been evident, where participants seek the approval of the researcher by trying to give the ‘correct’ answer [27]. Men often stated that they would intend to change their behaviours if requested. However, an ‘intention-behaviour gap’ may exist; this is a social construct that attempts to understand the psychological processes underlying whether or not intentions are translated into action, where what one says he or she plans to do, is not always translated into behaviour [28].

Analysis must be interpreted with caution, as those who had agreed to be interviewed for the current qualitative research may not be representative of the general prostate cancer population. Additionally, this research involved an exclusively White British population and the convenience sampling technique may have resulted in a non-representative sample. However, the men had undergone a range of treatments and varied in age, education level and occupation status. Thus, the data reported are believed to be representative of Caucasian men with prostate cancer and their partners, across the UK. Future research should aim to explore more diverse populations, particularly with regards to ethnicity. It should be noted that there are differences between the experience of surgery and radiotherapy, and that men were at different stages of their treatment pathway, and this could have resulted in differing responses based on treatment mode, for example when referring to incontinence. Despite this however, differences in responses were not always split depending upon treatment type, suggesting relevant similarities were evident across the two treatment types. Future research should consider approaching patients either prior to treatment or at a fixed time after treatment. Additionally, we did not systematically record the dietary or physical activity habits of the participants, or record their current BMI. This may have influenced their responses to the discussion; however it was felt that this level of quantitative data collection was not appropriate for a qualitative study. Finally, we note that two interviewers conducted the interviews, across different locations. This may have influenced the data collection process, however, both interviewers were experienced qualitative researchers, the interview schedule was used during data collection and data triangulation was carried out throughout the data processing and analysis period.

### Clinical implications

Lessons learnt can be implemented in clinical practice. The finding that men could be more likely to make changes if encouraged to do so by their clinician, is an ideal opportunity to engage with patients about such changes. Clinicians should be aware of potential barriers to behaviour change, possible solutions to such barriers should be discussed. The current sample were highly willing to participate in research, and clinicians should feel confident discussing research participation with their patients. These implications for practice are in line with the Living With and Beyond Cancer Programme [29], which resulted from the National Cancer Survivorship Initiative [29], and state that all cancer patients should have access to a holistic needs assessment, patient education and support, which should include post treatment management and the promotion of health and wellbeing, such as physical activity. With this support, new behaviours that are made may have a greater chance of lasting, and short-lived changes avoided [30].

## Conclusion

Generally, men facing a prostate cancer diagnosis are willing to make changes to their diet and physical activity, and this potential teachable moment should be embraced. Social support, tailored enjoyable interventions and background knowledge should be incorporated into an intervention package. Potential barriers should be pre-empted, and addressed in advance. Data illustrated that the proposed intervention would be well received in this population, with support for an individual physical activity intervention, provision of supplements and involvement of a partner throughout. Of note was the novel finding that modern technology and use of social media may not be suitable to all research populations, and thus the individual characteristics of the target population need to be considered. This research will be used to inform the development of a diet and physical activity intervention which is acceptable to prostate cancer patients who have undergone treatment. The research has additionally provided further information about the attitudes of Caucasian British men with prostate cancer, and their experiences of and preferences for different aspects of behaviour change, which can be implemented into clinical practice.
